# Workplace Violence against Nurses Working at Private Teaching Hospitals of Kathmandu: A Descriptive Cross-sectional Study

**DOI:** 10.31729/jnma.8555

**Published:** 2024-04-30

**Authors:** Sabina Dahal, Nishchal Devkota, Simran Pradhan, Rohan Jha, Hom Prasad Adhikari, Purna Laxmi Maharjan

**Affiliations:** 1 National Open College, Sanepa, Lalitpur, Nepal; 2 Vayodha Hospitals Private Limited, Balkhu Chowk, Kathmandu, Nepal; 3 Suvekchya International Hospital, Sitapaila, Kathmandu, Nepal

**Keywords:** *hospitals*, *nurses*, *violence*, *workplace violence*

## Abstract

**Introduction:**

Workplace violence in hospitals is a global concern and is considered as a major occupational hazard for all health care providers including the nurses. The aim of this study was to assess the status of workplace violence against nurses at hospitals in Kathmandu and determine the actions taken to investigate its cause.

**Methods:**

A descriptive cross-sectional study was conducted among a convenient sample of 100 registered nurses employed in Nepal Medical College and Teaching Hospital, and Kathmandu Medical College and Teaching Hospital. All eligible nurses who were willing to participate irrespective of their academic fulfilment, from all different shifts and of age below 45 years were included. Data were collected using a structured questionnaire and analysed using SPSS software. Ethical approval was taken from the Institutional Review Committee (IRC) of Nepal Medical College and Kathmandu Medical College.

**Results:**

Among 100 participants, the prevalence of workplace violence was 72 (72%) (62.13-80.52, 95% Confidence Interval). Verbal abuse accounted to 50 (69.44%), followed by physical violence accounting 17 (23.61%). Action was taken to investigate the causes of both physical violence 5 (29.41%) and verbal abuse 2 (4%) by the hospital administration 3 (60%) in physical violence and 2 (100%) in verbal abuse and police 2 (40%) in physical violence.

**Conclusions:**

The study reveals a troubling reality, as the vast majority of nurses reported experiencing various forms of violence in their workplace. So, addressing this issue immediately could protect nurses' well-being and ensure quality care which benefits both healthcare professionals and patients.

## INTRODUCTION

Workplace violence includes mistreatment, intimidation, or attacks endangering employees' health. Prevention involves clear policies, training, security measures, and promoting a positive workplace culture.^1^'^4^ National Institute for Occupational Safety and Health (NIOSH) defines it as any physical assault, threats, or verbal abuse at workplace.^5^ Postworkplace violence effects include loss of confidence, absenteeism, self-medication, and substance-abuse.^6^ Nurses experience a bidirectional relationship between nursing shortages, stress, and burnout.^7^

In Nepal, hospital-based descriptive cross-sectional study in Baglung and Pokhara found 64.9%,^2^ and 64.5% of nurses experienced violence, with verbal abuse being the highest at 61.5%.^8^ The Nepalese government has addressed working conditions and safety through the Labor Act 1992,^9^ and has passed "Health Professional and Health Institutions Protection" act in response to violence against medical professionals.^10^

This study was conducted to assess the prevalence of work place violence against nurses working in selected hospitals of Kathmandu Metropolitan.

## METHODS

This descriptive cross-sectional study was carried out among registered nurses employed in Nepal Medical College and Teaching Hopsital (NMCTH), and Kathmandu Medical College and Teaching Hospital (KMCTH) in Nepal. This study was conducted to find out the magnitude of workplace violence experienced by nurses in the selected health care settings of Kathmandu. The study was conducted from September 2021 to January 2022. Data were collected from the participant nurses who were currently working in the selected hospital using structured questionnaire which was adopted and modified as per the objectives of study from WHO/ILO/PSI/ICN survey questionnaire.^11^ The modified questionnaire was pre-tested in 35 nurses at one of the orthopaedic hospitals of Nepal.

Prior to conducting this research study, formal approval from World Health Organization (WHO) to adopt their structured survey questionnaire related to WPV formed in 2003 was taken. Ethical approval was taken from the Institutional Review Committee (IRC) Board (Reference Number: 007-078/79//2308202107) of NMCTH and KMCTH. The participants were informed regarding the study objectives, methods and expected benefits of this study. Written consent was taken from the participants willing to participate in this research study. Nurses were chosen on a first meet first basis for data collection at each hospital until the target number - 100 nurses from two teaching hospitals were reached. No pressure of any kind was applied to the individual to be a part of research, the personal identity and information provided by the subject was maintained confidential and protected by the law "Right to Privacy".

All eligible nurses who were willing to participate irrespective of their academic fulfilment, from all different shifts and of age below 45 years were included. Information related to socio-demographic variables such as participants age, marital status, work experience was taken.

Sample size of 100 was taken that was determined by using the formula


$$\begin{array}{ll} \text{n} & ={\text{Z}}^{2}\times \frac{\text{p}\times \text{q}}{{\text{e}}^{\text{2}}}\\ & =1.96^{2}\times \frac{0.56\times 0.43}{0.1^{2}}\\ & =92.5 \end{array}$$

Here,

n = minimum required sample size
Z = standard normal variate and equals 1.96 at a 95% Confidence Interval (CI)
p = prevalence conducted on WPV among nurses in teaching hospital in Nepal which is 56.47 %^12^
e = margin of error, 10%

As the minimum sample size was 92.5, a sample size of 100 was taken. As the sample was collected in two teaching hospitals only, with limited number of participants, a nonprobability convenient sampling method was used. Although workplace violence is a quite sensitive issue and nurses might not have opened up truthfully, they accepted to be a part of study as all participants filled the questionnaire. The data was coded and computed on a computer using the SPSS software program.

The descriptive analysis of the relevant variables was done and interpreted according to the objective of the study. The findings are presented in the tables and in narrative form.

## RESULTS

Among 100 respondents, 72 (72%) (62.13-80.52, 95% Confidence Interval) nurses faced a spectrum of violent occurrences in their workplace setting. Specifically, physical violence accounted for 17 (23.61%) cases, verbal abuse for 50 (69.44%) cases, bullying or mobbing for 1 (1.38%) case, and sexual abuse for 4 (5.55%) cases of reported instances. Out of 100 participants, the majority of the respondents were between the ages of 20 to 29 years with 50 (50%) belonging to the age category of 25-29 years followed by 39 (39%) belonging to the age group 20-24 years. More than half of the participants that is 64 (64%) were unmarried and all the participants were full time working female nurses. This was the data collected solely focused on two private teaching hospitals in Kathmandu. Out of the total respondents, 88 (88%) have had work experience of 1-5 years (Table 1).

**Table 1:** Personal and workplace data of respondents (n= 100).

Socio-demographic variables	n (%)
Age	
20-24	39 (39)
25-29	50 (50)
30-34	8 (8)
35-39	1 (1)
Above 40	2 (2)
Marital Status	
Single	64 (64)
Married	36 (36)
Work Experience	
1-5 years	88 (88)
6-10 years	8 (8)
11-15 years	2 (2)
16-20 years	2 (2)

Among all the 100 participants, 72 (72%) were the victims of workplace violence. Majority of them had experienced verbal abuse accounting 50 (69.44%), and it was done by all mentioned perpetrators which included patients 21 (42%), their relatives 13 (26%), staffs 8 (16%) and supervisor 8 (16%). Whereas in second place, 17 (23.61%) had experienced physical violence which was split roughly into four fifth and one fifth between patients; 13 (76.47%) and relatives of patients 4 (23.53%), but staff and supervisor were not involved at all. In case of sexual abuse, all perpetrators were staff members (Table 2).

**Table 2:** Perpetrators by type of violence (n= 72).

Perpetrators	Types of violence			
	Physical violence n (%)	Verbal Abuse n (%)	Bullying/ Mobbing n (%)	Sexual Abuse n (%)
Patient/ Client	13 (76.47)	21 (42)	-	-
Relative of patient	4 (23.52)	13 (26)	-	-
Staff member	-	8 (16)	1 (100%)	4 (100%)
Management/ Supervisor	-	8 (16)	-	-

The impact of violence was divided into two categories of frequency of disturbed thoughts of attack and hypervigilance after attack which was further broken down into level of impact (how bothered were they) ranging from not at all, a little bit, moderately, quite a bit up to extremely.

Following the incidents of physical violence, among 17 participants, 11 (64.71%) of them were a little bit bothered by the attack, quite a few 5 (29.41%) were moderately bothered and 1 (5.89%) was disturbed quite a bit but none of the victims were extremely bothered. Also, 6 (35.29%) of them were moderately alert, 5 (29.41%) each were extremely and a little bit alert and 1 (5.89%) was quite a bit alert.

In 50 cases of verbal abuse, affected victims depicted varied range of responses. More than half of them 29 (58%) were bothered a little bit, whereas 10 (20%) of them weren't bothered at all. In terms of vigilance after attack, almost half of them, 18 (36%), were a little bit alert (Table 3).

**Table 3:** Impact of violence to the victims.

Attributes	How much bothered are you? (Repeated, disturbing memories/ thoughts of attack)				How much super-alert have you become after an attack?			
	Physical Violence (n= 17) n (%)	Verbal Abuse (n= 50) n (%)	Bulling/ Mobbing (n= 1) n (%)	Sexual Abuse (n= 4) n (%)	Physical violence (n=17) n (%)	Verbal Abuse (n= 50) n (%)	Bulling/ mobbing (n= 1) n (%)	Sexual Abuse (n= 4) n (%)
Not at all	-	10 (20)	-	-	-	8 (16)	-	-
A little bit	11 (64.71)	29 (58)	-	-	5 (29.41)	18 (36)	-	-
Moderately	5 (29.41)	10 (20)	1 (100)	-	6 (35.29)	16 (32)	1 (100)	-
Quite a bit	1 (5.89)	1 (2)	-	4 (100)	1 (5.89)	1 (2)	-	-
Extremely	-	-	-	-	5 (29.41)	7 (14)	-	4 (100)

In this study, the actions taken to investigate the cause of violence were also studied. According to the findings, action was taken in case of 5 (29.41%) physical violence and 2 (4%) verbal abuse cases. Investigation was carried out mostly by hospital administration- 3 (60%) of physical violence and 2 (100%) of verbal abuse cases. Verbal warning was given to 3 (60%) of physical violence abusers and 2 (100%) of verbal abusers. In case of bullying and sexual harassment, no action was taken at all because they didn't feel comfortable. As per the respondents, reporting or taking any action against the abuser was pointless. This shows a trend of inaction in sensitive cases of workplace violence (Table 4).

**Table 4:** Action taken to investigate the cause of violence, investigator, and consequences for attacker/abuser (n= 72).

Was action taken to investigate the cause of violence?	Physical Violence n (%)	Verbal Abuse n (%)	Bullying/ Mobbing n (%)	Sexual Harassment n (%)
Yes	5 (29.41)	2 (4)		
No	10 (58.82)	40 (80)	1 (100)	4 (100)
Don't Know	2 (11.76)	8 (16)		
**If yes, who investigated?**				
Management	3 (60)	2 (100)		
Police	2 (40)			
**What were the Consequences?**				
Verbal warning	3 (60)	2 (100)		
Care discontinued	2 (40)			

## DISCUSSION

According to Asian researches, Taiwan had higher frequency of verbal abuse, followed by physical assault, bullying/mobbing, sexual and racial harassment,which aligned with our study (Physical Violence-23.61 %, Verbal Abuse-69.44%, Bullying/Mobbing-1.38%, Sexual Abuse-5.55%).^13^ Similar patterns were identified in Macau, where verbal abuse surpassed sexual and racial harassment.^14^ Bangladesh, on the other hand reported over 60% of non-physical violence experience.^14^ According to the studies conducted in Pokhara and Baglung of Nepal, almost 60% reported abuse, with highest being verbal abuse (64.9%), and lowest being sexual abuse (9%)which was much analogous to the current study. ^2,8^

Research showed higher prevalence of PVV (Patient Visitor Violence) in OPD, emergencies, post-ops, anaesthesia, ICUs and when dealing with patients above 65 years.^16^ Physical violence was more common in emergency, geriatric ward.^15^ Physical violence happened more in Anglo region whereas bullying and verbal abuse occurred more in Middle East.^16,17^ In UK, medical wards had the highest prevalence rate of WPV, 87% Israeli medical staff,^17^ and 75% of Jordanian emergency department had faced WPV.^19^ Our study also revealed 72% of the staff had faced some kind of workplace violence.

The psychological impact of workplace assault during studies have shown positive correlation of psychological disturbance and incidence of assault.^6,13^ Nurses exposed to verbal or physical abuse often experienced a negative psychological impact post incident.^6^ In a study done in health care workers in Taiwan about one-third to half of the victims of workplace violence suffered Post-traumatic Stress Disorder(PTSD).^13^ In this study as well, this idea has been reinforced, most of the respondents have been little bit mentally disturbed (Physical Violence - 64.71%, Verbal Abuse - 58%); with all respondents who had undergone bullying/mobbing responding moderately disturbing memories of the incident.

Respondents who were assaulted have also shown hyper-alertness at workplace after these incidents on average at least a little bit (Physical Violence-29.41%, Verbal Abuse-36%); whereas in case of bullying/ mobbing-100% reported being moderately super alert. ^20,21^ Super-alertness in workplace can be linked to fear of such incidents being repeated again. All sexual assault survivors responded as becoming extremely super-alert after such incidents (100%).

The data in this study showed a pattern of little faith in the system as no action was taken in most cases (Physical Violence - 58.82%, Verbal Abuse - 80%, Bullying/Mobbing- 100%, Sexual Harassment- 100%). Nurses were reluctant to address these types of incidents, worrying about repercussions of reporting. As per the respondents, reporting or taking any action against the abuser was pointless.^4^ This showed a need of systemic empowerment of nurses, a need for proper training regarding rights mentioned in policies while also creating an optimal environment for them to exercise said rights. A Nepali study conducted in 201, concluded that Nepal government should create zero tolerance policies against WPV, especially in the highly volatile healthcare sector.^10^

This study was carried out in a restricted number of teaching hospitals in Kathmandu metropolitan during a specific timeframe. Therefore, the results may not be applicable to a broader context. Despite choosing a relatively larger precision (allowable error) in our study, the sensitivity of the topic and the challenges associated with enrolling respondents, especially in a medical cohort such as nurses, still justify our approach. Additionally, the extent of violence cannot be measured in a quantitative study. Moreover, the investigation cannot precisely ascertain the details of how the incidents occurred and how they were addressed.

## CONCLUSIONS

The study highlights the growing prevalence of Workplace Violence (WPV) against nurses. The findings reveal a troubling reality, as the vast majority (more than two-thirds) of pooled nurses reported experiencing various forms of violence in their workplace. The most common type of abuse was verbal, followed by physical, sexual and mobbing/bullying. This escalating trend compromises not only nurses' well-being, but also the quality of care provided to patients. It is critical that healthcare professionals, politicians, and associations work together to create effective programs to reduce WPV. By addressing this issue immediately, we protect nurses' well-being and ensure quality care which benefits both healthcare professionals and patients.
